# The British Home for Incurables

**Published:** 1888-05-12

**Authors:** 


### "THE DANERIES."

### BRITISH HOME FOR INCURABLES.

WHAT may be considered *the* event of the present London season will be an Anglo-Danish Exhibition and Fête to be held in the Exhibition grounds, South Kensington, and which on Monday next will be opened by H.R.H. the Princess of Wales, in aid of the rebuilding of this national charity, which was the first institution in England to receive the patronage of her Royal Highness on her arrival in this country. By the courtesy of Mr. R. G. Salmond, the indefatigable secretary of the British Home for Incurables, we are informed that the lease of the present premises at Clapham will shortly expire, and that a new Home will have to be provided. A suitable freehold site can be obtained on which it is proposed to erect a building, not costly as to architectu al appearance, but replete with every modern improvement for the relief of those permanently afflicted. For this, however, a sum of £15,000 is required, and the managers of the "Daneries" are in the hope of being able to hand over to the charity this amount as the result of the exhibition. Great progress is being made in the preparations for the opening ceremony, and we are assured that everything will be completed by Monday.

The entrance hall is decorated with the Danish colours (red and white), and a particularly pleasing effect has been obtained by the suspension of coloured Wenham lamps from the galleries. In close proximity to the buildings is a theatre of large proportions, replete with stage, raised floor, and private boxes, wherein will be produced, under the direction of Mr. Savile Clarke, *tableaux vivants*, with musical interludes, illustrating the fairy lore of Denmark, immortalised by Hans Christian Anderssen. A good company has, we understand, been engaged for these representations, and the dresses have been designed by Chasemore. The scenery is the work of Mr. T. C. Banks, and the *tableaux* promise to be one of the great attractions of the exhibition. In the gardens, which will be laid out as a pleasant promenade, and illuminated by night, will be reproduced "Hamlet's Grave" and "Ophelia's Well," the former being an especially effective piece of artificial work. Under a rustic bridge flows a pellucid brook. At the opposite side of the grounds Mr. Hart is making a huge stalactite cavern. Near at hand is one of the chief features of the fête, namely, the Danish Village, with its picturesque windmill and inn. This village will be inhabited by peasants from Amager land dressed in the national costume of the district. During the exhibition two or more of the shops in the Danish Village will be utilised by a committee of ladies, over which H.R.H. the Princess Mary Adelaide, Duchess of Teck, is president, for a sale of work sent by friends of the charity for disposal for the benefit of the building fund.

A collection of works by Thorvaldsen will be gathered together, and there will also be a collection of the flora and fauna of Denmark and its dependencies.

The corvette *Dagmar*, which has been for some time icebound among the Norwegian fiords, has sailed from Denmark, having Prince Karl of Denmark, the second son of the Crown Prince, together with the second son of the King of Greece, among the naval cadets on board, who will take an official part in the opening ceremony. The sailors, it is understood, will form a guard of honour to H.R.H. the Princess of Wales and remain a short time in London.

Her Royal Highness will be received at the Albert Hall by H.R.H. the Duke of Cambridge, as president of the committee of patrons, and a reception committee, consisting of representatives of the Board of Management of the British Home for Incurables, the Directorate of the Exhibition, and the Danish community resident in London. The National Anthem will be sung by Madame Albani, and the Exhibition declared open by H.R.H. the Duke of Cambridge.

Purses of £5 5*s*. and upwards will also be received by her Royal Highness on behalf of the building fund from ladies and children. A Danish national song will then be sung by Mdlle. Otto Brönnum in Amager costume, and Mdme. Albani will give "Home, Sweet Home."

In addition to instrumental concerts, which will be given by the bands of both the Coldstream and Grenadier Guards, there will be constant open-air entertainments of a high-class character, and in unseasonable weather these entertainments will be given under cover.

On Monday evening, after the opening ceremony, H.R.H. the Duke of Cambridge will preside at a festival dinner, to be held in the Conservatory at South Kensington, in aid of the building fund, and we sincerely trust that this laudable exhibition will meet with the generous support of the public, which the energies and devotion of the directorate have a right to expect.

